# Evaluation of alfalfa inter-seeding effect on bahiagrass baleage fermentation and lactating Holstein performance

**DOI:** 10.1186/2049-1891-5-38

**Published:** 2014-07-24

**Authors:** Michael E McCormick, Kun Jun Han, Vinicius R Moreira, David C Blouin

**Affiliations:** 1 Southeast Region LSU Agricultural Center, 21549 Old Covington Hwy, Hammond, LA 70403, USA; 2 Louisiana State University School of Plant, Soil and Environmental Sciences, Baton Rouge 70803, USA; 3 Louisiana State University Agricultural Center, Southeast Research Station, P.O. Drawer 569, Franklinton 70438, USA; 4 Department of Experimental Statistics, Louisiana State University, Baton Rouge 70803, USA

**Keywords:** Alfalfa, Bahiagrass, Baleage, Corn silage, Lactating dairy cows

## Abstract

**Background:**

Previous research indicates that bahiagrass may be successfully conserved as baleage, but nutritive value is typically low for lactating dairy cows. The purpose of this study was to determine the effect of adding modest amounts of alfalfa forage (22%), achieved by inter-seeding alfalfa into an existing bahiagrass pasture, on baleage nutritive value and lactation performance of Holstein cows. Forage treatments employed were monoculture bahiagrass baleage (MBB; negative control), bahiagrass-alfalfa mixture baleage (BAB) and conventional corn silage (CCS; positive control). Thirty six mid lactation Holstein cows [34.8 ± 5.8 kg 3.5% fat-corrected milk and 112 ± 19 d in milk (DIM)] were stratified according to milk yield and DIM and assigned randomly to 1 of 3 forage treatments. Cows were trained to Calan feeding gates and were offered a common CCS-based TMR in a 10-d covariance period followed by a 42-d treatment feeding period.

**Results:**

The BAB contained more protein and less NDF than MBB (12.6 vs 10.3% CP and 71.8 vs 76.6% NDF). Diet DMI was similar for MBB and BAB (19.5 vs 21.6 kg/hd/d), but cows consumed more of the CCS diet (25.5 kg/hd/d) than either baleage-based diet. Cows offered BAB tended to produce more milk than cows offered MBB based TMR (28.4 vs 26.1 kg/hd/d), but both baleage diets generated less milk than CCS-based diets (33.1 kg/hd/d). Milk composition was similar across diets except for milk protein concentrations which were higher for CCS than either MBB or BAB diets; however, milk urea nitrogen (MUN) was lowest for cows fed CCS diets. Cow BW gain was higher for BAB than MBB implying that a portion of the higher energy contributed by the alfalfa was being used to replenish weight on these mid lactation cows.

**Conclusions:**

Data from this study indicate that alfalfa inter-seeded in bahiagrass sod that produces BAB with as little as 22% alfalfa may improve nutritive value compared to monoculture bahiagrass baleage and marginally improve lactation performance of Holstein cows. However, the CCS diet was vastly superior to either MBB or BAB-based diets for milk production.

## Background

Bahiagrass (*Paspalum notatum*, L.) is a common perennial forage base for many dairies in southern Louisiana and Mississippi, USA [1]. When properly managed, this forage is high yielding and persistent, particularly given the often harsh environmental conditions of the area. However, nutritive content of conserved bahiagrass is often below requirements of young growing animals and lactating dairy cattle [2]. In spite of its low nutritive value and water soluble carbohydrate content, bahiagrass may be successfully conserved as baleage [3].

Interseeding legumes into perennial grass pastures often increases animal growth and milk production above that of animals grazing pure grass stands [4,5]. Most of the research on interseeding legumes into grass pastures has been conducted in the Northeastern and Midwestern USA where conditions are favorable for legume production. Researchers in Louisiana attempted to inter-seed red clover (*Trifolium pretense*) into bermudagrass (*Cynodon dactylon*) and bahiagrass pastures for lactating dairy cattle [6]. They recorded a 10% (4 pounds FCM) increase in milk yield for cows grazing grass-clover pastures compared to those grazing grass alone. During the last decade, alfalfa varieties have been developed which are especially adapted for grazing in southern climates [7]. Research using these varieties has focused on interseeding (no-till drilled) in existing bermudagrass pastures [8,9]. Bermudagrass stand loss has been a concern with interseeding due to earlier spring emergence of alfalfa and shading of bermudagrass plants. Increasing alfalfa row spacing from 20 to 60 cm aided in maintaining the bermudagrass stand and reduced alfalfa seeding rate from 19 to 6.4 kg/ha. No information on overall forage quality was provided with these reports nor is information available on more traditional hay type (upright growing) alfalfa plants inter-seeded in summer perennial grasses. Research on inter-seeding alfalfa in existing bahiagrass stands and its effect on conserved forage nutritive value are not available. Though bahiagrass has a dense sod, it was speculated that a late fall interseeding of alfalfa would allow sufficient early spring alfalfa production to improve the nutritive value of the resulting bahiagrass-alfalfa bale silage crop. A lactation performance study was conducted to evaluate the nutritive value of subsequent bahiagrass-alfalfa baleage fed to lactating dairy cows. Monoculture bahiagrass served as a negative control and corn silage (*Zea Mays*, L.) was included as a positive control.

## Materials and methods

### Forage production, storage and sampling

All agronomic and animal procedures described in this report were conducted at the Louisiana State University Agricultural Center’s Southeast Research Station located approximately 8 km west of Franklinton, LA (Lat 30° 47’ 04” N and Lon 90 12’ 19” W). A 4.9 ha field of ‘Argentine’ bahiagrass (*Paspalum notatum*, var. *latiflorum*) was top-dressed with 4.5 Mg/ha dolomitic lime on May 25, 2006. On December 19, 2006 the bahiagrass was inter-seeded with 7.4 kg live seed/ha of ‘Amerigraze 702’ alfalfa (*Medicago sativa*, *L*.; America’s Alfalfa, Madison, WI) with a Bush Hog Model 9690 no-till drill (M & L Industries, Inc., Baton Rouge, LA). Alfalfa seeds were factory-coated with material containing a fungicide (metalaxyl-M) and rhizobia (*Sinorhizobium meliloti*) constituting 34% of planted seed weight. Alternating seed drops on the planter were closed to provide row widths of approximately 30 cm. In late February the inter-seeded bahiagrass-alfalfa was top-dressed with 140 kg K, 60 kg P, 28 kg S, 24 kg N and 4 kg B fertilizer per ha. Following an initial late-April hay harvest, the bahiagrass-alfalfa field was top-dressed with 125 kg K, 17 kg P, 15 kg N, 26 kg S and 3 kg B fertilizer per ha. A nearby (<300 m distant) 5.7 ha field of ‘Argentine’ bahiagrass was used as a negative control i.e. monoculture bahiagrass. Fertilizing and harvest schedules were similar to that of the inter-seeded field except lime was applied at 2.25 Mg/ha and fertilizer consisted of 449 kg/ha of a blended fertilizer containing 24% nitrogen, 10% phosphorus and 17% potassium.

On June 11^th^, 2007 at approximately 1000 h, the bahiagrass-alfalfa field (second harvest; 43 d regrowth) was harvested with a Kuhn model FC-300C disc cutter equipped with a flail-type conditioner (Kuhn North American, Inc., Broadhead, WI). The regrowth contained bahiagrass with less than 10% seed heads and 1/10-bloom stage alfalfa. One meter-length windrow samples were collected from four random locations within the field and separated into bahiagrass, alfalfa and weed fractions. Forage was allowed to wilt in 1.2 m windrows for approximately 24 h after which high-moisture forage was baled into 1.5 m × 1.2 m round bales using a model 568 John Deere baler equipped with a high moisture kit (John Deere, Inc., Cary, NC). Prior to wrapping, six randomly selected bales were weighed on a digitized platform scale (Rice Lake Weighing Systems, Rice Lake, WI) and core-sampled to a depth of 45 cm in four locations and forage was composted and stored on ice until processed in the Southeast Research Station Forage Quality Laboratory. Within 1.5 h post-baling, bales were individually wrapped (Ag Wrap, Ag Nations Products, Inc., Canton, OH) in six layers of 25-μm-thick white stretch film stretched 50% (Silage Wrap Sun Film, AEP Industries, Inc., Chino, Ca, USA). After wrapping, bales were moved to a well-drained location and stored outside on the ground. Hereafter, wrapped high moisture bales from the bahiagrass field inter-seeded with alfalfa will be designated as bahiagrass-alfalfa baleage (BAB).

The adjacent 5.7 ha field of monoculture bahiagrass was cut on June 12, 2007. Forage was managed in a manner similar to that described for the BAB. Windrow samples were handled as above and evaluated for bahiagrass and weed content. Hereafter, wrapped high moisture bales from the monoculture bahiagrass field will be referred to as bahiagrass baleage (MBB).

Corn silage was produced from Pioneer ‘31R87RR’ corn seed (Pioneer Hi-Bred International, Inc., Johnston, IA, USA) planted with a John Deere Maxi-Merge no-till planter (John Deere, Inc., Cary, NC, USA) in late March 2007 at a seeding rate of 64,200 plants ha. The non-irrigated corn was fertilized with 202 kg N/ha as urea, 31 kg/ha P and 88 kg/ha K at planting. Silage was harvested in mid-July at 1/3 milk line stage, chopped to 1.2 cm theoretical length and stored in a 2.7 m × 42.0 m silage bag (Ag Bag Model 9135) Ag Bag Systems, Inc., Astoria, OR, USA. Six green forage samples were collected every 7 m of bag length during silo filling and transported to the Southeast Forage Quality Laboratory for analysis. In future references within this report, this positive control forage will be referred to as conventional corn silage (CCS).

### Animal management

Thirty six mid lactation Holstein cows (34.8 ± 5.8 kg 3.5% fat-corrected milk (FCM) and 112 ± 19 d in milk (DIM)) were stratified according to lactation number (18 multiparous and 18 primiparous), DIM and milk yield and allotted to forage treatments. Cows were housed in a free stall barn equipped with Calan Gates (American Calan, Norwood, NH, USA) for measuring individual intake. Cows were trained to Calan Gates for 2 wk, after which they were subjected to a 10-d covariance period followed by a 42-d treatment feeding period. During the standardization period, all cows were fed a corn silage-based total mixed ration (TMR) containing 17.1% crude protein (CP), 22.7% acid detergent fiber (ADF) and 37.8% neutral detergent fiber (NDF) formulated to meet energy, CP, rumen undegradable protein (RUP) and mineral requirements for a 613 kg Holstein cow producing 39 kg FCM daily [10]. All animal procedures employed in this study were approved by the Louisiana State University Animal Use and Care Committee.

Milk weights were automatically recorded for each cow at each a.m. and p.m. milking using the AFI-Milk 2000 Information System (Germania Dairy Automation, Waunakee, WI, USA). Morning and evening milk sample composites were collected weekly, preserved with 2-bromo, 2 nitropropane-1, 3-diol and analyzed for fat, protein, lactose and urea N via infrared spectroscopy and SCC was determined via flow cytometry (Louisiana DHIA, Baton Rouge, LA, USA). Body weights and body condition score (BCS; 1 = extremely thin and 5 = extremely obese) [11] were recorded on two consecutive days at the beginning and end of the trial. Body condition scores were assigned by three independent observers.

On the morning of d 42, 10 mL of rumen fluid was collected pre-feeding from each animal via rumenocentesis [12]. Rumen fluid pH was measured immediately with an Orion model 230A portable pH meter (Orion Research Inc., Boston, MA) and the sample was then placed on ice until transported to the Southeast Research Station Forage Quality Laboratory. Five mL of rumen fluid was sterilized with 1.0 mL 25% meta-phosphoric acid and samples were centrifuged at 7,000 g in a refrigerated centrifuge (4°C), filtered through a 2.0 micron filter and stored at -20°C.

### Diet preparation and Lab analyses

Forages conserved as baleage (BAB and MBB) were ground with a flail type hay grinder (Model 2554 HayBuster, McConnell Machinery Co., Lawrence KS) into a vertical auger TMR mixer (Jaylor Model 3650, Orton, Ontario, Canada). A fresh bale of each treatment baleage was processed each morning between 0700 and 0830 h during the 42 d feeding period. Ground forage treatments were augured from the TMR mixer wagon onto a conveyor and into a Calan Data Ranger (American Calan, Inc., Norwood, NH, USA) whereupon concentrates were added to complete the experimental TMRs. Corn silage was handled in a manner similar to ground baleages. Cows were individually fed once per day between 0800 h and 0930 h to leave 10% or more orts.

Orts were collected and weighed prior to feeding each day. Ort and TMR grab samples were collected daily and stored at 4°C. These samples were composited weekly and used to determine TMR nutritive value and DM intake. Grab samples of ground baleage and corn silage were also taken daily at the conveyor, composited weekly and later analyzed for nutritive value in the Southeast Research Station Forage Quality Laboratory (Franklinton, LA, USA). A 1.0 kg sample of ground forage was collected weekly and manually evaluated for particle length (Table 1). During the second half of the study (wk 4–6), ambient and ort temperatures were recorded using a hand-held Raynger ST60 laser thermometer (Raytek, Inc., Santa Cruz, CA, USA). Ort temperatures were measured only for those cows leaving at least 5.0 kg fresh weight. Temperatures were taken from the approximate center of the ort TMR mass.

**Table 1:** **Chemical composition, fermentation characteristics and particle size of silage crops (% of DM)**^**1**^

	**Silage crop**^**2**^		
**Item**	**MBB**	**BAB**	**CCS**
DM	55.77 ± 13.03	51.60 ± 9.70	29.23 ± 1.13
CP	10.32 ± 0.38	12.64 ± 0.89	9.53 ± 0.46
ADF	42.18 ± 1.19	41.50 ± 0.60	26.55 ± 0.83
NDF	76.56 ± 1.73	71.80 ± 1.76	45.70 ± 1.94
IVTD^3^	68.12 ± 5.97	71.34 ± 3.54	80.61 ± 2.50
NE_L,_ Mcal/kg	1.23 ± 0.04	1.32 ± 0.06	1.56 ± 0.04
WSC^3^	2.22 ± 0.96	3.77 ± 1.05	10.26 ± 4.20
pH	5.71 ± 0.55	5.39 ± 0.32	3.76 ± 0.09
Lactate	0.73 ± 0.29	0.58 ± 0.12	3.47 ± 0.59
Acetate	0.24 ± 0.12	0.27 ± 0.05	4.78 ± 1.57
Propionate	0.00	0.00	0.69 ± 0.29
Butyrate	0.00	0.03 ± 0.01	0.05 ± 0.02
Isobutyrate	0.02 ± 0.01	0.00	0.00
Valerate	0.04 ± 0.10	0.03 ± 0.06	0.00
Isovalerate	0.01 ± 0.01	0.00	0.00
Calcium	0.32 ± 0.03	0.57 ± 0.07	0.15 ± 0.05
Phosphorus	0.17 ± 0.01	0.27 ± 0.02	0.87 ± 0.21
Potassium	1.49 ± 0.29	1.88 ± 0.12	0.96 ± 0.23
Magnesium	0.25 ± 0.05	0.23 ± 0.02	0.13 ± 0.01
Copper, ppm	4.00 ± 1.45	3.61 ± 1.66	2.29 ± 2.01
Manganese, ppm	236.1 ± 121.1	147.0 ± 63.0	35.38 ± 8.79
Zinc, ppm	25.71 ± 4.18	22.2 ± 3.65	24.63 ± 1.58
Post-ensiling particle size^4^		% of total	
< 1.0 cm	24.9 ± 14.4	22.5 ± 12.1	37.5 ± 29.0
1 to 7.4 cm	13.9 ± 3.5	13.2 ± 1.7	13.2 ± 4.4
7.5 to 14.8 cm	17.4 ± 2.0	25.9 ± 1.6	16.7 ± 6.9
14.9 to 22.2 cm	16.2 ± 4.9	22.3 ± 4.8	17.2 ± 10.6
22.3 to 29.6 cm	13. 1 ± 5.3	10.5 ± 4.3	8.6 ± 9.1
> 29.7 cm	14.4 ± 10.5	5.6 ± 5.1	6.8 ± 5.9

^1^Determined from forage samples collected each day and composited weekly (n = 7).

^2^*MBB* = monoculture bahiagrass baleage, *BAB* = bahiagrass-alfalfa baleage and *CCS* = conventional corn silage.

^3^*IVTD* = *in vitro* true digestibility; *WSC* = water soluble carbohydrates.

^4^Based on fresh samples collected biweekly and manually measured for particle length.

Weekly silage and TMR composite samples were weighed, dried at 55°C for 48 h and ground through a 1-mm screen. Ground samples were analyzed for DM and CP (micro Kjeldahl) according to AOAC [13] procedures. Sample ADF and NDF concentrations were determined using the detergent fiber methods of Van Soest et al. [14]. Sodium sulfite and amylase were added to NDF solutions before fiber analyses were conducted. *In vitro* digestibility (IVTD) of silage samples was determined by the two stage rumen fermentation-pepsin technique as described by Goering and Van Soest [15] and water soluble carbohydrate (WSC) concentrations were determined using the phenol-sulfuric acid procedure [16]. Volatile fatty acids (VFA) for silage and rumen fluid samples were measured via gas–liquid chromatography [17]. Rumen fluid lactic acid concentration was determined via the enzymatic conversion of L-lactate to pyruvate using the YSI 2700 Select Biochemistry Analyzer (YSI, Inc., Yellow Springs, OH).

Bahiagrass net energy was estimated by the equation: NE_L_ (Mcal/kg) = 2.2 × [1.085 – (0.0124 × ADF)] and corn silage by the equation: NE_L_ (Mcal/kg) = 2.2 × [1.044 – (0.0124 × ADF)] [3]. Concentrate mix NE_L_ was estimated based on NRC [10] estimates of NE_L_ for grain supplement ingredients [10]. Concentrations of Ca, K, Mg, Mn, Zn and Cu in silages and TMRs were determined by dry ashing, solubilizing in 20% HCl and analyzing via atomic absorption spectroscopy (Analyst 30, Perkin Elmer, Norwalk, CT) [13]. Phosphorus was determined by the molybdovanadate colorimetric method [13].

### Statistical analyses

Lactation performance data were statistically analyzed using the MIXED procedure of SAS Institute [18]. All daily intake and milk production data were reduced to cow-week means before statistical analysis. The model for the lactation performance data included forage treatment, week and forage treatment x week as fixed effects; cow nested within treatments as random effects; and cows by week as residual error effects. Performance data collected during the standardization period were used as covariables for the dependent variables. Treatment means were generated via Tukey-Kramer procedures and results are accompanied by the highest standard error. Statistical differences between means were declared at *P* < 0.05 and tendencies were reported at *P* > 0.05 and < 0.10.

## Results and discussion

### Forage agronomic and nutritive value assessment

Although alfalfa broke dormancy several weeks prior to bahiagrass, alfalfa constituted less than 10% of the forage mix in the initial late April harvest. However, at the second cutting on June 11, 2007 the alfalfa accounted for 22.3% of the forage mixture, bahiagrass accounted for 76.8% and weeds represented the remaining 1.5% of the forage mass (BAB treatment). The monoculture bahiagrass field contained 97.1% bahiagrass and 2.9% weeds at the second cutting (MBB treatment). The bahiagrass-alfalfa harvest used for the study generated forty five 1.5 m × 1.2 m round bales weighing an average of 659 kg fresh weight. Average DM yield for the second cutting was 3,235 kg/ha for BAB and 3,450 kg/ha for MBB treatment.

Chemical composition, fermentation characteristics and particle size of silage crops are presented in Table 1. The CP levels were low and NDF high for MBB (10.3 and 76.5%, respectively), but these concentrations are similar to values reported by Florida researchers for 5-wk bahiagrass regrowth [19]. Crude protein concentration in BAB was 22.5% higher and NDF was 7.3% lower than MBB, reflecting the higher nutritive value of alfalfa present in the BAB mixture [20]. *In vitro* digestibility and net energy for lactation of bahiagrass baleage were also improved by interseeded alfalfa. In general, research with tropical grass-legume mixtures indicates nutritive values similar to the ratios of the grass and legumes present in the mixture [21]. Corn silage *in vitro* digestibility and NE_L_ were considerably higher than those of either bale silage crop, though this was an exceptionally high-energy corn silage crop for dry land corn grown in southeastern Louisiana [2]. Poor bahiagrass baleage nutritive value i.e. low protein and high NDF, was also reported in previous baleage work at this unit [3], but NDF levels were higher in the present study likely due to the later stage of maturity of harvested bahiagrass.

Dry matter content in baleage crops MBB and BAB was similar and was well within the optimum range of 40-60% described by Muck [22]. Since DM differences between MBB and BAB were minimal, pH and fermentation characteristics were more likely influenced by forage composition than baleage moisture content. Residual WSC concentrations were much lower for baleage crops than corn silage and coupled with high pH indicate a restricted fermentation for MBB and BAB. A slight increase in silage WSC and decrease in pH was noted for BAB compared to MBB, but pH values for both baleage crops were above 5.0, the maximum pH threshold required for inhibition of undesirable clostridial fermentations [22]. In spite of this restricted fermentation, high levels of mold were not observed in bale interiors probably a result of relatively low baleage moisture concentrations [23] and airtight bale wrapping. Lactic acid was the predominant organic acid present in baleage extracts though concentrations were less than one fourth of that found (4% of DM) in high WSC-containing cool season grasses [24]. As McEniry et al. [23] noted, extent of baleage fermentation is often limited in low moisture forage crops. Similarly, Foster et al. [19] recorded bahiagrass baleage WSC of 3.72% post-ensiling and lactate concentrations equaled less than 1% of DM. However, Foster et al. [19] noted butyrate concentrations of 0.39%, whereas we were unable to detect any butyrate in our MBB silage. In fact, only nominal levels of VFAs were detected for either baleage crop with the exception of acetate whose average concentration was 0.24 and 0.27% for MBB and BAB, respectively. Overall, the pH, organic acid concentrations and nutritive values were quite similar between the two bahiagrass baleage crops with each data set indicating that bahiagrass is a forage crop low in WSC and overall nutritive value, which under the management conditions imposed, may none the less be successfully ensiled as baleage. Corn silage pH was typical of that recorded for well-preserved chopped whole plant corn (<4.0), but acetic acid concentrations were inordinately high (4.78% of DM) suggestive of hetero-fermenting bacteria dominant ensiling [24].

Post-grinding particle sizes for silage crops presented in Table 1 varied considerably between MBB, BAB and CCS (non-ground). More than 27% of the MBB forage exceeded 22 cm in length compared to 16.4 and 15.4% for BAB and corn silage, respectively. Lower levels of long particles in BAB than MBB suggest that alfalfa forage length was more efficiently reduced by grinding than bahiagrass forage as would be expected based on lower alfalfa NDF concentrations. In addition, nearly 50% of corn silage particles were less than 7.4 cm in length compared to only about 35% for the baleage crops. In spite of our best efforts to reduce baleage particle length via a hay grinder, baleage crop particle lengths remained substantially longer than those of corn silage during the feeding study.

Ingredient and chemical composition of experimental diets are presented in Table 2. Forages represented approximately 47.5% of DM for all experimental diets. Diet CP, ADF, NDF, and IVTD concentrations were similar for MBB and BAB. These data contradict the individual silage compositions in which CP and IVTD were higher and NDF was slightly lower for BAB than MBB (Table 1). These findings are in contrast to those of Titterton and Bareeba [21] who noted that nutritive value of tropical grass – legume mixtures was generally a reflection of individual nutrient composition and the ratio of grass to legume. In our study, windrow samples indicated about 22% alfalfa content in the mixed sward which should have led to higher BAB protein content than experienced based on forage and TMR analyses. This implies that actual legume contribution to BAB may have been lower than recorded or sampling procedures failed to generate representative samples. We did note considerable variation in field-wide alfalfa growth prior to forage harvest. This variation in percentage bahiagrass and alfalfa was also likely present within BAB bales. As expected, the corn silage-based TMR was lower in fiber fractions and higher in IVTD than either baleage-based TMR.

**Table 2:** **Ingredient and chemical composition of experimental diets (% of DM)**^**1**^

	**Diets**^**2**^		
**Item**	**MBB**	**BAB**	**CCS**
Ingredient composition			
Corn, grd	21.93	23.92	21.93
Soybean meal, 55%	17.74	15.73	17.75
Cottonseed	8.34	8.34	8.34
Mineral mix^3^	1.74	1.74	1.74
Sodium bicarbonate	0.89	0.89	0.89
Rumen inert fat^4^	0.85	0.85	0.85
SC^5^	0.63	0.63	0.63
Calcium carbonate	0.43	0.43	0.43
Forage	47.45	47.47	47.44
Chemical composition			
CP	16.38 ± 1.65	16.10 ± 0.93	17.07 ± 1.08
ADF	27.02 ± 1.65	26.90 ± 1.79	19.25 ± 2.62
NDF	50.30 ± 2.82	47.61 ± 3.21	32.11 ± 2.23
NE_L,_ Mcal/kg	1.41 ± 0.08	1.47 ± 0.10	1.62 ± 0.11
IVTD^6^	74.90 ± 2.16	74.30 ± 6.35	87.38 ± 3.57
Calcium	0.62 ± 0.08	0.67 ± 0.07	0.69 ± 0.13
Phosphorus	0.34 ± 0.03	0.36 ± 0.02	0.45 ± 0.02
Potassium	1.77 ± 0.18	1.77 ± 0.23	1.35 ± 0.16
Magnesium	0.30 ± 0.04	0.29 ± 0.03	0.29 ± 0.02
Copper, ppm	16.40 ± 3.75	14.29 ± 2.82	17.54 ± 5.51
Manganese, ppm	222.4 ± 89.9	145.9 ± 40.2	87.4 ± 28.8
Zinc, ppm	89.0 ± 11.3	82.3 ± 12.8	100.3 ± 6.6

^1^Chemical composition of total mixed rations determined from samples collected daily and composited weekly (n = 7).

^2^MBB = mono culture bahiagrass baleage-based diet, BAB = bahiagrass-alfalfa baleage-based diet and CCS = conventional corn silage-based diet.

^3^Mineral and vitamin mix containing 23% Ca, 3% P, 7% Mg, 6% K, 3.0% S, 15 mg Co/kg, 650 mg Cu/kg, 50 mg I/kg, 1200 mg Mn/kg, 2700 mg Zn/kg, 18 mg Se/kg, 300,000 IU Vitamin A/kg, 30,000 IU Vitamin D/kg, and 1,500 IU Vitamin E/kg.

^4^MegaLac rumen inert fat manufactured by Church and Dwight, Inc., Princeton, NJ.

^5^Saccharomyces cerevisiae yeast product sold by Diamond V Mills, Inc. Cedar Rapids, IA.

^6^IVTD = *in vitro* true digestibility.

### Lactation performance and rumen characteristic*s*

Lactation performance data are presented in Table 3. Alfalfa inclusion in the bahiagrass baleage did not improve DM intake though cattle offered BAB showed numerically higher (10.5%) diet consumption. Cattle fed CCS-based TMR consumed 24.2% more (*P* < 0.05) DM than the average consumed by the cows fed baleage-based TMRs. Expressed as a percent of final body weight, DMI was 3.16, 3.35 and 3.74% of BW for MBB, BAB and CCS, respectively. Intake for the corn silage based diets was similar to that predicted by NRC [10], but cattle consumed the baleage-based diets at considerably lower levels than predicted. Differences in DMI between baleage diets and CCS were likely related to the higher digestibility, and smaller particle size of corn silage compared to the baleage treatments (Table 2). Also, NDF intake as percent BW was 1.65% for baleage-based diets compared to only 1.25% for corn silage-based diets. Long particle length and high NDF concentrations have been shown to limit DMI for both tropical [25] and cool season grasses [26].

**Table 3:** Lactation performance of Holstein cows fed total mixed diets containing monoculture bahiagrass baleage (MBB), bahiagrass-alfalfa baleage (BAB) or conventional corn silage (CCS)

	**Silage crop**			
**Item**	**MBB**	**BAB**	**CCS**	**SE**
Cows	12	12	12	
DM intake, kg	19.51^a^	21.56^a^	25.50^b^	0.75
CP intake, kg	2.86^a^	3.20^ab^	3.48^b^	0.24
NDF intake, kg	9.81^a^	10.26^a^	8.18^b^	0.85
Milk yield, kg^1^	26.11^a^	28.44^b^	33.12^c^	0.71
3.5% FCM, kg	27.58^a^	29.83^a^	35.26^b^	1.20
Fat,%	3.87^a^	3.95^a^	3.83^a^	0.12
Fat, kg	1.00^a^	1.09^a^	1.28^b^	0.06
Protein,%	3.08^a^	3.04^a^	3.24^b^	0.04
Protein, kg	0.89^a^	0.89^a^	0.95^b^	0.01
Lactose,%	4.64^a^	4.68^a^	4.78^b^	0.02
Lactose, kg	1.37^a^	1.38^a^	1.40^a^	0.01
MUN, mg/dL	16.80^a^	15.76^a,b^	14.31^b^	0.53
SCC × 1,000	304^a^	209^a^	221^a^	89
FE^2^, kg fcm/kg DMI	1.49^a^	1.42^a^	1.38^a^	0.06
Initial BW, kg	611.17^a^	611.26^a^	657.12^a^	21.50
Final BW, kg	616.90^a^	643.38^a^	681.29^a^	21.60
BW gain, kg	5.73^a^	32.12^b^	24.17^ab^	4.71
Initial BCS	2.83^a^	2.63^a^	2.64^a^	0.15
Final BCS	2.78^a^	2.67^a^	3.03^a^	0.13
BCS change	-0.05^a^	0.04^a^	0.39^b^	0.11

^a, b, c^Means in a row with different superscripts differ significantly (*P* < 0.05).

^1^Milk yield for BAB tended to be higher than MBB (*P* < 0.10). Milk yield for CCS was higher than either MBB or BAB (*P* < 0.05).

^2^*FE* = feed efficiency.

**Figure 1:**
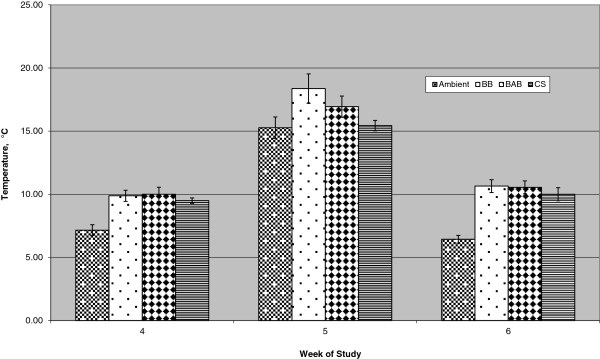
**Ambient and ort temperatures for bahiagrass baleage (BB), bahiagrass-alfalfa baleage (BAB) and conventional corn silage (CCS).** Values are weekly means and standard deviations.

Secondary fermentation i.e. diet heating, may also have influenced MBB consumption. From wk 4 to 5 of the study, minimum ambient temperature increased from 7.3 to 15.2°C which increased TMR refusal temperature 8.1 degrees (from 10.0 to 18.1°C) for MBB compared to a 5.5 degree increase (from 9.7 to 15.2°C) for CCS (Figure 1). Higher pH and lower VFA concentrations, especially lower acetic acid in baleages compared to CCS may have allowed more rapid proliferation of molds and yeasts which led to more extensive TMR heating and lower palatability [27].

Milk yield tended to be higher (*P* < 0.10) for cows offered BAB than MBB (28.44 vs 26.11 kg/hd); however, when expressed on a 3.5% FCM basis milk yields between the two baleage crops were similar. Since the percentage improvement in milk yield with alfalfa introduction was about 9% for both actual and FCM, lack of statistical difference between the two baleage treatments for FCM was likely due to higher variability in milk fat concentration as evidenced by a SE for actual milk yield of 0.71 compared to 1.20 kg/hd for FCM. Cows fed CCS-based diets produced substantially more (*P* < 0.05) actual and FCM than cows fed either BAB or MBB-based diets as expected based on differences in diet NDF and NE_L_ concentrations (Table 2). Actual milk yields and NRC [10] predictions for energy allowable milk were similar among diets. Feed efficiency averaged 1.43 kg FCM/kg DMI and did not differ with dietary treatment. Research studying inclusion of cool season perennial legumes in warm-season perennial grass silage diets is scant, but substitution of red clover for bermudagrass pasture or ryegrass silage indicated a substantial improvement in DMI and FCM yield but not in feed efficiency [6,28].

Percentage milk fat concentration did not differ among dietary treatments. Since both baleage diets contained higher NDF concentrations and longer particle lengths, it was anticipated that baleage treatments would generate higher milk fat concentrations than CCS-based diets. The fact that they did not may partially be explained by the higher DMI for CCS, but may also be related to inherently low NDF digestibility for the bahiagrass-based diets [19]. However, rumen fluid analysis demonstrated that acetate, the major precursor for milk fat synthesis, was present in higher concentrations for MBB than the other two silages (Table 4). These inconsistencies may be related to time of rumen fluid sampling (4 h fast) that favored larger particle sizes and slower fiber digestion rates for bahiagrass baleage-based TMRs vs corn silage-based TMRs.

**Table 4:** **Rumen fluid characteristics of Holstein cows fed total mixed diets containing monoculture bahiagrass baleage (MBB), bahiagrass-alfalfa baleage (BAB) and conventional corn silage (CCS)**^**1**^

	**Silage crop**			
**Item**	**MBB**	**BAB**	**CCS**	**SE**
pH	6.40^a^	6.61^a^	6.55^a^	0.11
Lactate	0.02^a^	0.03^a^	0.05^a^	0.01
Acetate	55.23^a^	39.60^b^	42.30^b^	3.11
Propionate	13.69^a^	9.20^b^	11.83^ab^	0.99
Butyrate	0.36^a^	1.08^a^	0.18^a^	0.26
Isobutyrate	9.02^a^	6.04^a^	6.30^a^	1.13
Valerate	17.90^a^	10.15^a^	14.06^a^	2.54
Isovalerate	0.12^a^	0.12^a^	0.54^a^	0.25
Total VFA	96.20^a^	66.19^b^	75.21^b^	5.75
A:P^2^	4.08^ab^	4.34^a^	3.67^b^	0.13

^a, b, c^Means in a row with different superscripts differ significantly (*P* < 0.05).

^1^Rumen fluid collected via rumenocentesis on d-42 prior to a.m. feeding (4 h fast).

^2^Acetate to propionate ratio.

Percent milk protein and lactose did not differ among cows fed BAB or MBB, but cows offered CCS generated higher (*P* < 0.05) levels of milk protein and lactose than either baleage crop. Higher milk protein and other component concentrations for CCS may be related to greater microbial protein synthesis and or enhanced dietary N utilization due to more of easily fermentable carbohydrate in the rumen than baleage-based diets. Milk urea nitrogen concentrations, though similar between BAB and MBB, were higher for baleage-based diets than CCS diets (16.3 vs 14.3 mg/dL) which may again suggest poorer N utilization by cows fed the baleage-based diets. Differences in MUN between bahiagrass and corn silage TMR were not likely due to differences in total dietary N intake as CCS diet was higher in CP intake than MBB and BAB (Table 3), but more likely differences were related to higher soluble N concentrations for the baleage crops compared to corn silage [29].

Body weight and condition score changes were generally positive, as expected for cows that averaged 162 DIM at study conclusion (Table 3). The BW gain was higher (*P* < 0.05) for cows on BAB than MBB (32.1 vs 5.7 kg/h/d) suggesting that a portion of the higher energy and numerically higher DMI for BAB may have been partitioned toward BW gain rather than milk production. Cows consuming MBB had the lowest BW gain and, on the average, experienced slight losses in BCS. Differences in BW gain between BAB and CCS were small, but BCS change was 0.35 and 0.44 units higher (*P* < 0.05) for CCS than either BAB or MBB, respectively. Again, higher DMI for CCS allowed cows to begin replenishing body condition earlier than experienced by cows receiving baleage-based diets.

## Conclusion

In conclusion, modest levels of alfalfa (22%) in bahiagrass-alfalfa mixture fed in this study improved forage energy and protein concentrations and tended to improve actual milk yield compared to monoculture bahiagrass forage. Neither MBB nor BAB was consumed as readily as CCS-based TMR by mid-lactation Holstein cows and cows offered CCS diets produced 23.0% more FCM than the average of cows offered MBB or BAB-based diets. Never the less, inter-seeding alfalfa into bahiagrass pastures may serve as a means of improving bahiagrass pasture and stored forage nutritive value.

## Abbreviations

MBB: Monoculture bahiagrass; BAB: Bahiagrass-alfalfa balaeage; CCS: Conventional corn silage; DIM: Days in milk; BW: Body weight; FCM: Fat-corrected milk; TMR: Total mixed ration; SCC: Somatic cell count; MUN: Milk urea nitrogen; DMI: Dry matter intake; FE: Feed efficiency; CP: Crude protein; ADF: Acid detergent fiber; NDF: Neutral detergent fiber; NRC: National Research Council; WSC: Water soluble carbohydrates; IVTD: *In vitro* true digestibility; VFA: Volatile fatty acids; BCS: Body condition score.

## Competing interests

The authors declare that they have no competing interests.

## Authors’ contributions

MM conceived the trial, generated forage treatments, conducted the feeding experiment and drafted the initial manuscript. KJH managed forage production and lab analyses; VM was instrumental in project development, animal training and animal data collection. DB participated in the design of the study and conducted all statistical analyses. All authors read the manuscript, participated in revisions and approved the final manuscript.
